# Prefrontal-Parietal White Matter Volumes in Healthy Elderlies Are Decreased in Proportion to the Degree of Cardiovascular Risk and Related to Inhibitory Control Deficits

**DOI:** 10.3389/fpsyg.2017.00057

**Published:** 2017-01-26

**Authors:** Pedro P. Santos, Paula S. Da Silveira, Fabio L. Souza-Duran, Jaqueline H. Tamashiro-Duran, Márcia Scazufca, Paulo R. Menezes, Claudia Da Costa Leite, Paulo A. Lotufo, Homero Vallada, Maurício Wajngarten, Tânia C. De Toledo Ferraz Alves, Patricia Rzezak, Geraldo F. Busatto

**Affiliations:** ^1^Laboratory of Psychiatric Neuroimaging, Institute and Department of Psychiatry, Universidade de São PauloSão Paulo, Brazil; ^2^Center for Interdisciplinary Research on Applied Neurosciences (NAPNA), University of São PauloSão Paulo, Brazil; ^3^Department and Institute of Psychiatry, University of São PauloSão Paulo, Brazil; ^4^Department of Preventive Medicine, Faculty of Medicine, University of São PauloSão Paulo, Brazil; ^5^Center of Research in Mental Health Population, University of São PauloSão Paulo, Brazil; ^6^Laboratory of Magnetic Resonance in Neuroradiology, Institute and Department of Radiology, University of São PauloSão Paulo, Brazil; ^7^Department of Internal Medicine, Center for Clinical and Epidemiologic Research, University of São PauloSão Paulo, Brazil; ^8^Department of Cardiopneumology, Heart Institute, General Hospital of University of São Paulo Medical SchoolSão Paulo, Brazil; ^9^Laboratory of Clinical Neurophysiology, Institute of Psychiatry, University of São Paulo Medical School (IPq-HC-FMUSP)São Paulo, Brazil

**Keywords:** voxel-based morphometry (VBM), framingham risk factor, cognition, APOEε4 allele, MRI imaging

## Abstract

Cardiovascular risk (CVR) factors may be associated with poor cognitive functioning in elderlies and impairments in brain structure. Using MRI and voxel-based morphometry (VBM), we assessed regional white matter (WM) volumes in a population-based sample of individuals aged 65–75 years (*n* = 156), subdivided in three CVR subgroups using the Framingham Risk Score. Cognition was assessed using the Short Cognitive Performance Test. In high-risk subjects, we detected significantly reduced WM volume in the right juxtacortical dorsolateral prefrontal region compared to both low and intermediate CVR subgroups. Findings remained significant after accounting for the presence of the APOEε4 allele. Inhibitory control performance was negatively related to right prefrontal WM volume, proportionally to the degree of CVR. Significantly reduced deep parietal WM was also detected bilaterally in the high CVR subgroup. This is the first large study documenting the topography of CVR-related WM brain volume deficits. The significant association regarding poor response inhibition indicates that prefrontal WM deficits related to CVR are clinically meaningful, since inhibitory control is known to rely on prefrontal integrity.

## Introduction

Cardiovascular risk factors (CVRF), including dyslipidemia, diabetes, smoking, hypertension and obesity, are highly prevalent in elderly populations and are associated with deficits in cognitive performance (Mortamais et al., 2014; Pierdomenico et al., 2015).

In recent years, several magnetic resonance imaging (MRI) studies have demonstrated the presence of macro-structural brain changes related to the severity of CVRF in groups of elderly subjects. These include silent brain infarcts (Smith et al., 2015) and findings of reduced gray matter volumes in the brain, most often involving frontal, temporal and parietal regions (de Toledo Ferraz Alves et al., 2011; Hayden et al., 2014). In addition, elderly individuals may present an excess of white matter (WM) hyperintensities, thought to reflect microvascular injuries (Söderlund et al., 2003; Gouw et al., 2011; Barker et al., 2014). Such lesions, most often located in frontal WM regions (Maillard et al., 2012) are significantly related to the degree of CVRF (Markus et al., 2005; Kearney-Schwartz et al., 2009; Williams et al., 2010; Rostrup et al., 2012; Homayoon et al., 2013) and are usually associated with impaired cognitive functioning (Knopman et al., 2001; Au et al., 2006; Debette et al., 2011).

The use of MRI also allows volumetric measurements of WM tracts in the aging human brain, and reduced WM volumes may reflect either primary local impairments of the structural integrity of WM tracts or Wallerian degeneration secondary to abnormalities in adjacent gray matter areas (O'Brien, 2014; Ito et al., 2015). A few MRI studies to date have investigated the presence of abnormal WM volumes in proportion to the severity of CVRF in samples of middle-aged or elderly subjects. These studies have often reported reduced whole-brain or regional WM volumes in elderly individuals with hypertension (Raz et al., 2003) or type 2 diabetes (Last et al., 2007; Chen et al., 2012; Moran et al., 2013), as well as variable findings of increased (Walther et al., 2010) or reduced (van Bloemendaal et al., 2016) WM volumes in direct proportion to increases in body-mass index. Regional WM volume changes in these MRI studies have been most often reported in the frontal lobe (Raz et al., 2003; Last et al., 2007; Walther et al., 2010; Chen et al., 2012; Moran et al., 2013), as well as in temporal (Walther et al., 2010; Chen et al., 2012; Moran et al., 2013) and parietal regions (Walther et al., 2010; van Bloemendaal et al., 2016).

One limitation of the above-cited MRI investigations of WM volumes relates to the lack of concurrent assessment of the presence of the epsilon4 allele of the apolipoprotein E gene (APOEε4), which may be a predictor of volume variations of both gray matter and WM compartments in the human brain (Wishart et al., 2006; Yokoyama et al., 2015; Dowell et al., 2016) The presence of the APOEε4 allele is known to be associated with elevated cholesterol levels (Bennet et al., 2007) and may also be related to other CVRF, such as type II diabetes (Janson et al., 2004). Another limitation relates to the fact that previous MRI studies investigating WM volumes have most often evaluated the isolated impact of single CVRF (Last et al., 2007; Gunstad et al., 2008; Paul et al., 2008; Chen et al., 2012); however, in a high proportion of elderly individuals CVRF appear in combination (Razay et al., 2007), and separate risk factors may display distinct effects on WM volumes in the same subjects (Walther et al., 2010; van Bloemendaal et al., 2016). In a population-based MRI study of elderly individuals aged 65 to 75 years (de Toledo Ferraz Alves et al., 2011), the use of the Framingham Risk Score (FRS), a composite measure that takes into account multiple risk factors (such as age, gender, blood pressure, smoking status, total cholesterol and high-density lipoprotein cholesterol levels, as well as the presence of diabetes) (Wilson et al., 1998), allowed our group to detect significant findings of regional gray matter loss in proportion to the degree of cardiovascular risk (CVR) (de Toledo Ferraz Alves et al., 2011). The FRS has been widely used in epidemiological studies of elderly populations (Jeerakathil et al., 2004; Seshadri, 2006; Seshadri et al., 2006).

In the present MRI investigation, we assessed the presence of WM volume abnormalities in relation to the degree of CVRF in our previously described population-based sample of elderly individuals (de Toledo Ferraz Alves et al., 2011) using the FRS. Based on the findings of previous MRI investigations of WM changes associated with CVRF (Raz et al., 2003; Last et al., 2007; Walther et al., 2010; Chen et al., 2012; Maillard et al., 2012; Moran et al., 2013), we predicted that reduced WM volumes in elderly subject with higher FRS scores would be seen particularly in frontal, temporal and parietal lobe regions. Also, we predicted that reduced WM volumes in subjects with higher CVR would be directly related to impaired cognitive performance. Finally, we sought to investigate the degree to which the relationship between CVRF and reduced WM brain volumes would be influenced by the presence of the APOEε4 allele.

## Methods

### Study sample

This retrospective MRI study received approval from the local Committee for Ethics and Research (CAPPesq) of the Faculty of Medicine, University of Sao Paulo (# 0450/05). Written consent was obtained from all subjects.

The study sample was selected from a community based database of elderly individuals recruited for the Sao Paulo Aging and Health (SPAH) study, the characteristics of which have been described in detail elsewhere (Scazufca et al., 2008). In that epidemiological investigation, all residents aged 65 or above of pre-defined census sectors of an economically disadvantaged area of Sao Paulo were contacted to undergo a cognitive evaluation following the protocol developed by the 10/66 Dementia Research Group (Prince et al., 2003; Scazufca et al., 2008). This protocol, developed for use in cross-cultural studies, included a structured neurological assessment, a structured cardiological evaluation, the Geriatric Mental State (GMS) (a standardized psychiatric interview), and the Community Screening Instrument for Dementia (CSI-D). A composite cognitive algorithm (COGSCORE) (Scazufca et al., 2008) was generated combining: an item-weighted summary score from the participant's 32-item CSI-D cognitive test; the Consortium to Establish a Registry for Alzheimer's Disease (CERAD) animal naming verbal fluency task; and the modified CERAD 10 word-list learning task with delayed recall (Copeland et al., 1986). All SPAH subjects were also assessed in regard to their CVR using the FRS, a composite index comprising five clinical factors (age, blood pressure, diabetes mellitus, smoking status, and cholesterol levels) (Grundy et al., 1998; Wilson et al., 1998; Coryell et al., 2005). Subjects recruited for the present MRI study were subdivided into three subgroups according to their degree of CVR (10-year risk) as follows: high-risk, FRS >20%; medium-risk, FRS 10–19%; and low-risk, FRS <9% (Grundy et al., 1999).

From the initial SPAH databank (*n* = 2072), we excluded: all subjects with a diagnosis of dementia using COGSCORE ratings (*n* = 105); those aged above 75 years at the time of recruitment for MRI scanning (*n* = 996); those with a history of major neurological disorders (such as epilepsy and Parkinson's disease) or lifetime diagnosis of major depressive disorder according to the International Statistical Classification-10th revision criteria (ICD-10) (*n* = 52); and those with missing clinical data (*n* = 107). This led to the identification of 812 potentially eligible individuals. Telephone contacts were then made with potentially eligible subjects to invite them to take part in this brain imaging study, to check for the presence of contra-indications for MRI scanning and evidence of cardiovascular disease (ie: history of cardiac surgery or use of cardiac stents, pacemakers, aneurysm clips), and to screen out anyone who had a previous history of head trauma with any loss of consciousness or loss of memory for events immediately before or after the accident for as long as 24 h. We were unable to successfully contact 103 subjects. From those who were reached, we excluded 206 subjects (132 females and 74 males) who fulfilled the above exclusion criteria, leaving us with a total pool of 503 subjects who could be invited to undergo the brain imaging session. Due to budget constraints, only 306 individuals were invited to participate in the MRI investigation; 52 of those refused to take part in the study, thus resulting in a total of 254 dementia-free elderly subjects aged between 66 and 75 years (female/male [134/114]) who underwent MRI scanning. A total of 68 subjects were excluded due to the presence of: cognitive deficits that were disproportionally greater than what would be expected for the SPAH population; brain vascular malformations; at least one silent brain infarct (i.e., silent stroke and lacunar infarcts); meningioma; or artifacts during MRI scanning. Finally, we excluded 30 individuals with missing data regarding APOE genotype, cognitive performance and/or incomplete cardiovascular composite data. Thus, the final study sample comprised a total of 156 cognitively preserved elderly subjects aged between 66 and 75 years (78 females and 78 males). In order to briefly document the cognitive performance of subjects on the day of MRI scanning, all individuals underwent a short (approximately 10 min) evaluation using the Short Cognitive Performance Test (SKT) validated in Brazil by Flaks et al. (2006). The SKT affords a rapid evaluation of overall cognitive performance as well as in specific domains, such as memory, attention and automatic inhibition, with higher scores indicating more severe cognitive impairment (Lehfeld and Erzigkeit, 1997; Lehfeld et al., 1997). Thus, measures of cognitive performance used in the present investigation comprised: total SKT scores; SKT-based attention, inhibition and memory subscores. Also, IQ estimates were obtained in all subjects with the Wechsler Abbreviated Scale of Intelligence (WASI) (Wechsler, 1999).

Magnetic resonance imaging (MRI) datasets were acquired using a 1.5 T General Electric Signa LX CVi scanner (Milwaukee, WI, USA) with a quadrature transmit/receive head coil with AC-PC alignment using the following acquisition protocol: (a) an axial T2 multiecho sequence of 100 slices with repetition time (TR) of 2800 ms and echo time (TE) between 25 and 250 ms with a total of 10 echo trains per excitation, a flip angle (FA) of 90 degrees, 240 mm field of view (FOV), 5 mm slice thickness, number of excitations (NEX) of 1 and an acquisition matrix of 256 × 192 mm; (b) a T2 axial FLAIR sequence of 20 slices with TR of 10002 ms and TE of 109.44 ms, a FA of 90 degrees, 240 mm FOV, 5 mm slice thickness, 6.5 mm spacing between slices, NEX of 1 and an acquisition matrix of 256 × 192 mm; (c) a Spoiled Gradient Echo (SPGR) sequence of 124 contiguous slices with TR of 12.1 ms and TE 4.2, a FA of 15 degrees, 240 mm FOV, 1.5 mm slice thickness and an acquisition matrix of 256 × 192 mm; (d) an Axial T2 sequence of 15 slices with TR of 4200 ms and TE 12.5, a FA of 90 degrees, 280 mm FOV, 5 mm slice thickness, 6.5 mm spacing between slices and an acquisition matrix of 256 × 192 mm. All MRI scans were examined by experienced radiologists unaware of the study aims, in order to identify the presence, number and location of silent brain lesions. Infarcts were detected as low-signal-intensity lesions on the SPGR sequence and hyperintense lesions on the T2-weighted images. Vascular lesions with 3 to 15 mm in diameter were classified as lacunae.

The two single nucleotide polymorphisms (SNPs) rs429358 and rs7412 that determine the three APOE isoforms (ε2, ε3, and ε4) were genotyped using methods detailed previously (de Toledo Ferraz Alves et al., 2011). Individuals were considered positive for APOE ε4 genotype if presenting at least one ε4 allele, including both heterozygous and homozygous carriers.

### Image processing: voxel-based morphometry

Imaging data (SPGR sequence) were processed using Statistical Parametric Mapping, Version 8 (SPM8, Wellcome Trust Centre of Neuroimaging, London, United Kingdom), implemented in MATLAB R2008a (MathWorks, Sherborn, MA). First, all anatomical images were reoriented; the mm coordinate of the anterior commissure matched the origin xyz (0, 0, 0), and the orientation approximated the Montreal Neurological Institute (MNI) space. Then, all images were segmented and classified into gray matter, WM and cerebrospinal fluid compartments using the unified segmentation routine implemented in SPM8, which provides both the native space versions and Diffeomorphic Anatomical Registration using Exponentiated Lie algebra (DARTEL) imported versions of the tissues (Ashburner and Friston, 2005). A customized template was created from the subjects using the DARTEL protocol (Ashburner, 2007; Matsuda et al., 2012). Subsequently, the deformation field was applied to the segmented images. Finally, the images created in the previous step were standardized to MNI space, re-sliced to 1.5 × 1.5 × 1.5 mm voxels and smoothed using an 8-mm full width at half maximum (FWHM) Gaussian kernel. The total WM volumes were obtained from the modulated segmented images and were given by the total number of voxels within the WM compartment of each subject. The choice to use a 8 mm Gaussian filtering size in the current study was based on the fact that such degree of smoothing provides an optimal degree of increment in signal-to-noise ratio and conformation of MRI data to a normal distribution (thus allowing the use of parametric tests in subsequent statistical comparisons). Also, this smoothing procedure compensates for some of the data loss incurred by spatial normalization (Salmond et al., 2002; Mechelli et al., 2005).

### Statistical analyses

#### Clinical and demographic data

Statistical comparisons of the three FRS subgroups in terms of demographic and genotype data were carried using the Statistical Package for Social Sciences (SPSS) for Windows (17.0 version). Chi-square tests were used for categorical variables, whereas ANOVAs were carried out for continuous variables. Levels of statistical significance were set at *p* < 0.05, two-tailed.

#### Between-group comparisons of neuroimaging data

Voxelwise comparisons of regional WM volumes were carried out between the 3 FRS subgroups using an ANCOVA model. Total WM volume, gender, age and years of education were included as confounding variables. Also, in order to investigate whether our VBM findings were influenced by APOE gene variations, we repeated the ANCOVA including presence of the APOEε4 allele as an additional covariate. Only voxels with values above an absolute threshold of *p* = 0.05 to differentiate gray matter and WM from other tissues were included in the analyses.

The statistics for each voxel of the whole WM compartment were transformed to Z scores and displayed as statistical parametric maps (SPMs) in standard MNI space at an initial threshold probability of Z > 3.09 (*p* < 0.001, uncorrected for multiple comparisons) and an extent threshold of 50 contiguous voxels. Initially, we inspected the SPMs resulting from the ANCOVAs using a statistical threshold of *p* < 0.05, familywise error (FWE)-corrected for multiple comparisons over the whole brain. However, the FWE-based whole-brain correction for multiple comparisons is highly stringent, increasing the risk of type II errors (Lindquist and Mejia, 2015). Therefore, we also conducted a region-of-interest based search for significant findings over frontal, temporal and parietal WM regions, as these were predicted *a priori* to show more prominent WM volume differences in proportion to the degree of CVR. This was carried out with a statistical threshold of *p* < 0.05, corrected for multiple comparisons over the search volume of each region-of-interest (minimum cluster size of 50 contiguous voxels), using the small volume correction (SVC) approach (Worsley et al., 1996). Significant ANCOVA findings were followed-up with two-group *post-hoc t*-tests. For the SVC-based analyses, WM regions-of-interest were spatially delineated by applying separate 20-mm radius spheres over dorsolateral prefrontal (DLPFC), orbitofrontal and lateral temporal WM areas on the two brain hemispheres, guided by the MRI Atlas of Human White Matter (Mori et al., 2008). The centroids of these spheres were defined visually, based on anatomical knowledge extracted from the MRI Atlas of Human White Matter (Mori et al., 2008) and the Talairach and Tournoux system. Centroid *x,y,z* coordinates for each sphere (right and left hemispheres) and search volumes were as follows: DLPFC area = 29,25,25 (9140 voxels) and −29,25,25 (8881 voxels); orbitofrontal area = 24,47,11 (7855 voxels) and −24,47,11 (7629 voxels); and lateral temporal area = 39,−39,14 (9596 voxels) and −39,−39,14 (9338 voxels). In addition, a parietal WM region-of-interest was spatially delineated using a 30-mm radius sphere with centroid coordinates of 25,−33,35 (30747 voxels) and −25,−33,35 (30873 voxels) for the right and left hemispheres, respectively.

In addition to the above between-group comparisons, voxelwise correlation analyses were performed between regional WM volumes and SKT results, always including total WM volume, gender, age and years of education as confounding covariates. Also, in order to statistically assess whether CVRF influenced on regional WM volume–cognitive performance relationships, we included “FRS subgroup status” as a further predictor variable in the analyses investigating the presence of significant correlations between WM volumes and cognitive performance score across the subgroups with low, medium and high CVR, searching for voxels where there might be significant differences in the pattern of WM volume-cognition correlations between the three subgroups. Such analyses were carried out for total SKT scores as well as separately for memory, attention and automatic inhibition subscores.

When reporting significant findings of all voxel-based analyses above, we converted the MNI coordinates of voxels with maximal statistical significance using the Talairach and Tournoux system (Brett et al., 2002).

## Results

### Demographic, genetic and cognitive characteristics of the sample

Table 1 presents the demographic, cognitive and APOE genotype characteristics for the whole sample (*n* = 156). A high proportion of individuals in the overall sample had very low levels of education (4 years or less, *n* = 112, 71.79% of the entire sample), reflecting the fact that all subjects were recruited in the same, economically disadvantaged catchment area of Sao Paulo, Brazil.

**Table 1:** **Demographic, cognitive and APOE characteristics of the study subgroups divided according to their cardiovascular risk using the Framingham Coronary Heart Disease Risk profile index**.

	**Low risk (*n* = 40)**	**Intermediate risk (*n* = 61)**	**High risk (*n* = 55)**	**Statistical test**	**Statistical significance**
Male/Female	8/32	23/38	47/8	*X*^2^ = 45.743	0.001
Mean age (±SD) in years	70.35 (2.28)	70.07 (2.30)	70.60 (2.39)	*F* = 0.762	0.468
Mean years of education (±SD)	4.65 (3.40)	5.26 (4.04)	4.15 (3.41)	*F* = 1.346	0.263
APOEε4 positive/negative	9/31	17/44	12/43	*X*^2^ = 0.676	0.713
Estimated IQ	78.7 (12.89)	76.15 (8.57)	76.62 (10.18)	*F* = 0.779	0.461
Total SKT score (±SD)	5.98 (4.31)	6.62 (3.95)	5.25 (4.20)	*F* = 1.580	0.209
Memory subscore	0.9 (1.12)	0.85 (1.09)	0.78 (1.01)	*F* = 0.147	0.863
Attention subscore	5.07 (3.82)	5.77 (3.64)	4.47 (4.01)	*F* = 1.672	0.191
Inhibition subscale (±SD)	1.3 (1.15)	1.52 (1.10)	1.13 (1.05)	*F* = 1.897	0.154

*APOEε4, apolipoprotein epsilon 4 allele; FRS, Framingham Coronary Heart Disease Risk; IQ, intelligence quotient; SD, standard deviation; SKT, Short Cognitive Performance Test; X^2^, chi-square test; F, F-test*.

We observed a gender imbalance across the three FRS subgroups (*p* = 0.001), with a predominance of women in the low-risk subgroup and a greater prevalence of men in the high-risk subgroup. There were no other significant between-group differences either in regard to demographic or APOE genotype data (Table 1).

The results of cognitive comparisons between the 3 subgroups are also displayed in Table 1. There were no significant between-group differences in any of the SKT-based measures or in regard to estimated IQ (Table 1).

### Between-group comparisons of WM volumes

The mean total volume obtained from the segmented WM images was equal to 463.14 ± 43.13 milliliters in the elderly sample investigated. There were no significant differences in total WM volumes between the 3 subgroups (ANCOVA, *F* = 4.24, *p* = 0.16).

**Figure 1:**
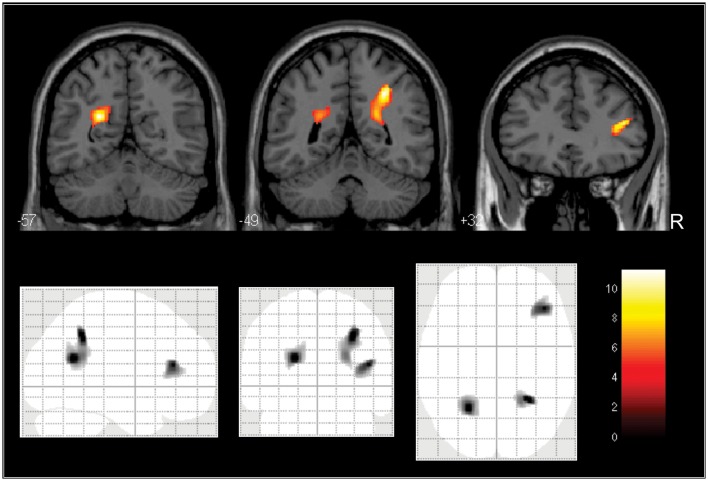
**Voxelwise WM volume comparisons between the 3 subgroups divided according to their degree of cardiovascular risk, with gender, age and years of education as cofounding variables**. At the bottom, glass-brain projections show three foci of between-group WM volume difference (in the right juxtacortical dorsolateral prefrontal WM and bilaterally in deep parietal regions). For display purposes, the statistical parametric map was filtered at *p* < 0.01 (uncorrected for multiple comparisons) with a minimum cluster extent of 250 voxels. At the top, the frames highlight (in yellow) the three foci of between-group WM volume difference overlaid on coronal brain slices spatially normalized into an approximation to the Talairach and Tournoux stereotactic atlas, located bilaterally in deep parietal regions (first and second frames) and in the right juxtacortical dorsolateral prefrontal WM (third frame). All findings retained statistical significance when we used the small-volume correction approach (*p* = 0.05, family-wise error corrected for multiple comparisons). *Post-hoc* two-group comparisons showed significantly reduced WM volume in the high-risk subgroup in comparison to the low-risk and intermediate-risk subgroups in the three regions (see statistical details for these comparisons in Table 2).

Regarding the VBM analyses, the inspection of the SPM for the ANCOVA comparing regional WM volumes between the 3 FRS subgroups showed three localized voxel clusters of between-group difference, situated, respectively, in the right juxtacortical DLPFC WM and bilateral deep parietal WM regions. The location of these three clusters is shown in Figure 1, filtered at a statistical threshold of *p* < 0.01 (uncorrected) for display purposes. No statistical significance was attained with the use of FWE-correction for multiple comparisons over the whole brain; however, when the SVC approach was used, statistical significance (*p* < 0.05, FWE-corrected) was attained for all three clusters, including: the right juxtacortical DLPFC WM (*F* = 9.79, *Z* = 3.72, 89 voxels, FWE-corrected *p* = 0.039), the left deep parietal region (*F* = 11.21, *Z* = 4.02, 132 voxels, FWE-corrected *p* = 0.041) and the right deep parietal region (*F* = 11.26, *Z* = 4.03, 96 voxels, FWE-corrected *p* = 0.039). Table 2 provides the results of the *post-hoc* two-group comparisons using the SVC-approach, which showed significantly reduced WM volume in the high-risk subgroup in comparison to the two other subgroups in the right DLPFC area and bilaterally in the parietal region.

**Table 2:** **Two-group comparisons of regional white matter volumes in brain regions where differences were predicted ***a priori*****.

**Brain region**	**Size of cluster^a^**	**Peak Z score^b^**	**Coordinates x, y, z^c^**	**P_FWE_ corrected^d^**
**GREATER WM VOLUME IN THE LOW RISK SUBGROUP COMPARED TO THE HIGH RISK SUBGROUP**				
Right juxtacortical dorsolateral prefrontal region	170	3.75	36, 31, 14	0.027
Right deep parietal region	108	3.95	30, −46, 37	0.041
**GREATER WM VOLUME IN THE INTERMEDIATE RISK SUBGROUP COMPARED TO THE HIGH RISK SUBGROUP**				
Right juxtacortical dorsolateral prefrontal region	220	4.15	42, 31, 17	0.020
Left deep parietal region	380	4.20	−20, −53, 22	0.017
Right deep parietal region	578	4.30	30, −46, 37	0.011
**GREATER WM VOLUME IN THE LOW RISK SUBGROUP COMPARED TO THE HIGH RISK SUBGROUP INCLUDING APOEε4 AS A CONFOUNDING VARIABLE**				
Right juxtacortical dorsolateral prefrontal region	190	3.73	36, 31, 14	0.025
Right deep parietal region	112	3.96	30, −46, 37	0.040
**GREATER WM VOLUME IN THE INTERMEDIATE RISK SUBGROUP COMPARED TO THE HIGH RISK SUBGROUP INCLUDING APOEε4 AS A CONFOUNDING VARIABLE**				
Right juxtacortical dorsolateral prefrontal region	218	4.13	42, 31, 17	0.021
Left deep parietal region	365	4.18	−20, −53, 22	0.019
Right deep parietal region	557	4.28	30, −46, 37	0.013

APOEε4, apolipoprotein epsilon 4 allele; WM, white matter;

a *Number of contiguous voxels that surpassed the initial threshold of p < 0.001 (uncorrected) in the statistical parametric maps*.

b *Z scores for the voxel of maximal statistical significance*.

c *Talairach and Tournoux (1998) coordinates of the voxel of maximal statistical significance within each cluster*.

d *Statistical significance after correction for multiple comparisons using the small-volume correction approach; inferences were made at the level of individual voxels (FWE, family-wise error correction)*.

### Influence of APOEε4 allele on WM volumes

Similar results as reported above were obtained when we repeated the ANCOVA comparing regional WM volumes across the whole brain between the 3 FRS subgroups including presence of the APOEε4 allele as covariate. There were only three voxel clusters of between-group difference, again located on the right juxtacortical DLPFC WM and bilaterally on the deep parietal region. No statistical significance was attained with the use of FWE-correction for multiple comparisons over the whole brain (*p* < 0.05), but when the SVC approach was used the three sites of between-group WM volume difference retained statistical significance: the right DLPFC region (*F* = 9.80, *Z* = 3.72, 90 voxels, FWE-corrected *p* = 0.039), the left deep parietal region (*F* = 10.94, *Z* = 3.96, 116 voxels, FWE-corrected *p* = 0.050) and the right deep parietal region (*F* = 11.20, *Z* = 4.02, 97 voxels, FWE-corrected *p* = 0.041). As shown in Table 2, *post-hoc t*-tests showed that WM volume in those three brain regions was reduced in the high-risk subgroup in comparison to the two other subgroups when the effect of the presence of the APOEε4 allele was covaried for.

Regardless of CVRF, we found no significant differences in total WM volumes between APOEε4 allele carriers (*n* = 38) and non-carriers (*n* = 118) (ANCOVA, *F* = 1.97, *p* = 0.46). There were neither significant regional WM volume differences when we compared APOEε4 allele carriers (*n* = 38) and non-carriers (*n* = 118).

### Correlations between regional WM volumes and cognitive performance

There were no statistically significant results when we investigated if cognitive deficits were associated with global WM loss by calculating (using SPSS) partial correlation coefficients between total WM volume and: SKT total scores; SKT attention sub-scores; SKT inhibition subscores; or SKT memory subscores (*r* < 0.30, *p* > 0.82). The same analyses were repeated separately for the three FRS subgroups and yielded no statistically significant results either.

Regarding the VBM analyses, the whole-brain voxelwise search for correlations between regional WM volumes and cognitive performance resulted on no significant findings for either of the 4 cognitive indices evaluated.

**Figure 2:**
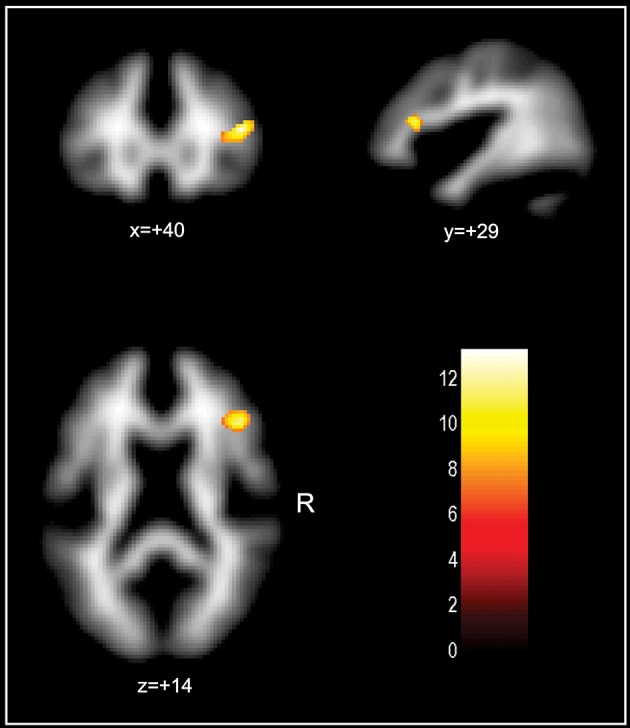
**Voxelwise analysis investigating the presence of significant correlations between white matter (WM) volume and inhibition performance score across the subgroups with low, medium and high cardiovascular risk, searching for voxels of significant difference in the pattern of WM volume-cognition correlations between the three subgroups (with total WM volume, gender, age and years of education were as confounding covariates)**. Findings were overlaid on transaxial brain slices spatially normalized into an approximation to the Talairach and Tournoux stereotactic atlas, filtered at *p* < 0.001 (uncorrected for multiple comparisons) with a minimum cluster extent of 50 voxels. The figure highlights (in yellow) one focus of interaction located in the right juxtacortical dorsolateral prefrontal WM (243 voxels, small-volume corrected for multiple comparisons, *p* = 0.003). The *post-hoc* inspection of this result indicated that there was a greater degree of inverse correlation between WM volume and inhibition performance in the right prefrontal region in the high-risk group relative to the low-risk group.

Finally, the analyses including “FRS subgroup status” as a predictor variable in order to statistically assess whether CVRF influenced on WM volume–cognitive performance relationships across the three subgroups showed one focus of interaction located in the right juxtacortical DLPFC WM region for the analysis of SKT-based inhibition subscores (*F* = 13.30; coordinates = 43,29,17 243 voxels, P_FWE_ = 0.003 SVC-corrected for multiple comparison). This finding is displayed in Figure 2. The *post-hoc* inspection of this result indicated that there was a greater degree of inverse correlation between WM volume and inhibition performance in the right prefrontal region in the high-risk group relative to the low-risk group. There were no other significant interaction findings for the other cognitive test results.

## Discussion

To the best of our knowledge, this is the first population-based voxelwise morphometric MRI study of elderly individuals investigating the presence of WM volume variations using the FRS score as a composite measure of CVR, in a sample free from dementia, MCI and silent gross cerebrovascular lesions. Voxelwise analyses of regional WM volumes allow assessment of the topographical distribution of WM changes associated with CVRF across the whole brain, thus providing complementary information to the more commonly reported macro-structural MRI investigations of WM hyperintensities (Smith et al., 2010; Gebeily et al., 2014; Nunley et al., 2015), gross cerebrovascular lesions (Launer et al., 2015; Petrou et al., 2016) and gray matter volume abnormalities (Freedman et al., 2015; Kharabian Masouleh et al., 2016) related to CVRF. Findings of our between-group comparisons indicated that elders with high CVR present WM volume deficits localized to the frontal and parietal lobes. Moreover, the results of our correlation analysis with cognitive performance suggest that changes in WM volumes related to CVRF are clinically meaningful, since a greater degree of significant correlation between reduced WM volume in the DLPFC region and worse performance in response inhibition (a cognitive operation widely known to rely on the integrity of the frontal cortex (Picton et al., 2007) was detected in the high-risk group. These findings provide support to the view that some of the cognitive deficits detected in elderly subjects (free from dementia or silent brain infarcts) may be mediated by WM abnormalities related to high CVR (Prins and Scheltens, 2015).

Our findings of reduced WM volume in association with high CVR were selectively located in the right juxtacortical DLPFC and bilateral deep parietal WM regions, with no between-group differences detected in any other brain region or in total WM volumes. Together with the predictable prefrontal location of CVR-related WM volume deficits, the finding of volume difference specifically in the deep parietal WM is also consistent with the results of previous studies investigating WM volume changes in relation to CVRF (Walther et al., 2010; van Bloemendaal et al., 2016). The parietal lobe location of CVR-related volume deficits is also expected given that parietal areas are anatomically connected to the prefrontal cortex via large WM tracts, ie. the superior longitudinal fasciculi.

At the chosen threshold of *p* < 0.001 for initially filtering the ANOVA statistical parametric map, the three clusters of between-group difference (respectively in the right DLPFC and bilateral parietal regions) were detectable with sizes of approximately 100 to 150 voxels each. Such localized, modestly sized findings are not to be considered unexpected, since we compared groups of individuals of the same age and with no history of neurological or other disorders that could affect the central nervous system. Our pattern of results suggests a predilection for vascular-related WM abnormalities to be located in the frontal lobes and parietal regions in healthy elderly individuals (Tullberg et al., 2004). In previous morphometric MRI studies that investigated WM volumes in relation to isolated CVRF, findings have been more widespread, involving the temporal lobe and other brain regions (Walther et al., 2010; Chen et al., 2012; Moran et al., 2013; van Bloemendaal et al., 2016). The absence of findings in additional WM regions in the present study is unlikely attributable to type II errors related to sample size, as the population-based sample studied herein is larger than the groups evaluated in most previous studies (Walther et al., 2010; Chen et al., 2012; van Bloemendaal et al., 2016). Given previous suggestions that separate CVRF may display distinct effects on WM volumes (Walther et al., 2010; van Bloemendaal et al., 2016), our results may indicate that when a measure of combined CVR is used, findings of selective frontal and parietal WM volume abnormalities stand out with greater statistical significance.

The right hemispheric location of prefrontal WM volume changes in our study is consistent with the hemispheric predilection of vascular-related findings in some previous imaging studies of the aging brain (Ito et al., 2008). The resent results are also consistent with our own previous investigations of regional cerebral blood flow in elders with heart failure (Alves et al., 2005) and regional gray matter volumes in the same elderly sample reported herein (de Toledo Ferraz Alves et al., 2011). It is difficult to explain readily such right hemispheric predilection, and it is not likely that the right hemisphere would be more vulnerable to vascular-related brain lesions. In fact, there is empirical evidence suggesting that both ischemic stroke and atherosclerotic plaques are commoner on the left hemisphere (Naess et al., 2006; Hedna et al., 2013; Selwaness et al., 2014). From the large elderly population, potentially available for our investigation, we excluded subjects with a history of stroke as well as those with silent brain infarcts detected by MRI scanning. It is therefore plausible that there was a selection bias toward the inclusion of more subjects with right-sided vascular-related brain changes in our study, while a greater proportion of elderlies with predominantly left-sided vascular-related abnormalities might have been excluded due to the worse outcome of such left-sided brain changes toward the emergence of stroke or silent brain infarcts.

The presence of the APOEε4 allele influences on brain volumes (Wishart et al., 2006; Yokoyama et al., 2015; Dowell et al., 2016) as well as being associated with elevated cholesterol levels (Bennet et al., 2007) and possibly other CVRF, such as type II diabetes (Janson et al., 2004). Therefore, any differences between our subgroups in regard to this gene polymorphism might exert a significant influence on our patterns of WM volume changes related to CVR. Indeed, it has been recently shown that vascular-related WM micro-structural changes as assessed in MRI studies using diffusion-tensor imaging (DTI) methods may be exacerbated among APOEε4 carriers (Wang et al., 2015). However, the frequency of the APOEε4 allele was not different between the 3 FRS subgroups in our study. Moreover, the pattern of between-group prefrontal differences in WM volumes remained unchanged when APOEε4 was added as a confounding variable, and no differences in WM volumes were detected between APOEε4 carriers and non-carriers regardless of FRS. Thus our results suggest that macro-structural WM volume changes associated with CVRF in non-demented elderlies may not necessarily be exacerbated by the presence of the APOEε4 allele.

The histological/molecular bases of the volumetric WM decrements detected in elders with high CVR in the present study remain to be elucidated. Ischemic microvascular lesions (presumably more frequent in elders with high CVRF) could reduce the local volume of WM matter in the brain (Ikram et al., 2008; Launer et al., 2015). Such kind of microvascular pathology has been previously shown to underlie brain WM hyperintensities in studies combining *in vitro* MRI and *post mortem* neuropathological assessments (Thomas et al., 2002, 2003, 2015; Tham et al., 2011). Frontal WM tracts are known to be particularly susceptible to the effects of local microvascular pathology in the brain (Craggs et al., 2014a,b). Alternatively, WM volume reductions could reflect microstructural WM changes secondary to neuronal degeneration in gray matter regions connected by large WM tracts (Gold et al., 2012; Haroutunian et al., 2014; Kawachi and Nishizawa, 2015). In a previous VBM analysis on the same elderly sample, we found lower gray matter volumes in temporal and parietal neocortices in proportion to the degree of CVR as assessed by FRS scores (de Toledo Ferraz Alves et al., 2011). The region where we found frontal WM volume deficits in the present VBM investigation correspond to the location of the *fasciculus uncinatus* and *superior longitudinal fasciculus*, which interconnect the temporal and parietal cortices to the frontal lobe (Catani and Thiebaut de Schotten, 2008; Thiebaut de Schotten et al., 2014).

Our findings of an association between volume of right juxtacortical frontal WM and inhibitory control abilities measured by the SKT inhibition score are consistent with the already well-stablished relationship of the right DLPFC with cognitive control abilities (Wang et al., 2016). One previous study showed that executive function skills were affected by the presence of CVRF, such as midlife hypertension and high waist-height ratio (Wolf et al., 2007). The findings presented herein are consistent with those of such previous study, since inhibitory control is known to be an executive function subdomain. As previously stated, the FRS includes potential CVRF other than hypertension and obesity. Thus, we add to the current literature by showing a relationship between WM abnormalities in the DLPFC and inhibitory control abilities in patients with greater FRS ratings.

In addition to the predicted location of CVR-related WM volume deficits in the prefrontal region, the additional findings specifically located in the deep parietal WM are consistent with previous investigations of WM volume changes in relation to CVRF (Walther et al., 2010; van Bloemendaal et al., 2016). The parietal lobe location of CVR-related volume deficits are also expected as parietal areas are anatomically connected to the prefrontal cortex via the superior longitudinal fasciculus. Parietal areas anatomically connect to frontal brain areas via superior longitudinal fascicles. Hence, reduction in both regional WM volumes in the high-risk group is not totally unexpected at all.

The results reported herein should be interpreted with caution given the methodological limitations of the study, related to our approach for image acquisition and analysis. First, we did not conduct automated, quantitative assessments of WM hyperintensities (Caligiuri et al., 2015); during segmentation routines for VBM analyses using SPM, WM hyperintensities could potentially reduce the local number of voxels classified as WM (Anbeek et al., 2004). Second, the spatial normalization step in the VBM methodology (whereby images are warped to conform to a standardized anatomical space) may be prone to inaccuracies which are more likely to occur when the degree of brain structural inter-subject variations is wider (as in the case of MRI datasets of elderly individuals) (Radanovic et al., 2013). Finally, our MRI data acquisition protocol did not include a diffusion tensor imaging (DTI) sequence, which would have afforded detailed information on the micro-structural characteristics of WM tracts in the brain. Recent DTI investigations with non-demented elderly subjects have suggested the presence of WM integrity abnormalities in proportion to the degree of CVR (Segura et al., 2009, 2010).

In conclusion, this study reported significantly reduced prefronto-parietal WM volumes in direct proportion to the severity of CVR in a population-based sample of elderly subjects free from dementia and other neuropsychiatric disorders. Also, we found an inverse relationship between cognitive (inhibitory control) performance and right DLPF WM volume. Future multimodal MRI studies with large elderly samples (including volumetric measurements, quantitative assessments of WM hyperintensities and DTI acquisitions in the same individuals) are warranted in order to extend the findings reported herein. Such multimodal MRI approach has been employed recently with success in studies of disorders, such as Alzheimer's disease (AD), affording complementary information of relevance that expands knowledge about the pathophysiology of brain neurodegenerative changes in elderly life (Radanovic et al., 2013).

## Author contributions

PS: Data analyzes and have written the paper. PD: Data collection. FS: Data analyzes QA. JT: Data collection. MS: Creator of the Sao Paulo Ageing and Health Study (SPAH). PM: Epidemiological analyzes for SPAH. CD: Neuroimaging acquisition. PL: Clinical data. HV: Genetic data. MW: Cardiological data. TD: Manager. PR: Senior researcher. GB: Senior researcher.

### Conflict of interest statement

The authors declare that the research was conducted in the absence of any commercial or financial relationships that could be construed as a potential conflict of interest.
